# A patient with osteoarthritis and cardiovascular disease presenting with bilateral hip pain: a case report

**DOI:** 10.4076/1757-1626-2-8823

**Published:** 2009-08-24

**Authors:** Jennifer Yanow, Marco Pappagallo

**Affiliations:** Mount Sinai School of Medicine, Department of Anesthesiology1 Gustave L. Levy Place, New York10029 USA

## Abstract

**Introduction:**

The pharmacological management of osteoarthritis normally begins with the administration of acetaminophen or a nonselective nonsteroidal anti-inflammatory drug. However, acetaminophen may not be efficacious in all patients, and nonsteroidal anti-inflammatory drugs may be associated with gastrointestinal and cardiovascular adverse effects.

**Case presentation:**

A 79-year-old Caucasian man with bilateral hip pain was diagnosed with osteoarthritis of the hip. His past medical history included three prior myocardial infarctions, the most recent one occurring 2 years ago. He is taking aspirin 81 mg once daily. Despite no history of ulcer disease or bleeding, he has been reluctant to take nonsteroidal anti-inflammatory drugs, and because of his cardiac history there is a contraindication to cyclooxygenase-2-specific inhibitors. He was started on naproxen 220 mg twice daily, with the proton pump inhibitor omeprazole 20 mg once daily. Upon follow-up at 4 weeks, his hip pain had decreased from a rating of 7 (on a ten-point scale) to 5 on his left side and from 5 to 2 on his right side. The patient began a course of physical therapy in conjunction with a regimen of naproxen 440 mg in the morning and 220 mg at night, plus the omeprazole and acetaminophen 650 mg twice daily. He reported no gastrointestinal effects.

**Conclusion:**

The addition of a proton pump inhibitor to nonsteroidal anti-inflammatory drug therapy can reduce the risk of peptic ulcer bleeding by ≥80%, making the incidence of gastropathy the same as with cyclooxygenase-2-specific inhibitors. The fact that naproxen is not associated with an increased risk of acute myocardial infarction made it an appropriate choice for this patient.

## Introduction

Osteoarthritis (OA) is the most common joint disorder in the United States and one of the leading causes of disability in the elderly. Approximately 5% of the population over the age of 65 years has radiographic evidence of OA of the hip, and nearly 200,000 total hip replacements are performed annually [1]. There is no cure for OA, but many pharmacologic options exist for managing its symptoms.

## Case presentation

A 79-year-old Caucasian retired physician presented to the office with a chief complaint of bilateral hip pain that had been present for approximately 2 years but had become increasingly bothersome over the previous 3 months. He described the pain as dull and achy and located anteriorly in both hips with occasional radiation to the groin. At its most severe the pain was rated as a 7 on a ten-point scale in the left hip and as a 5 in the right hip. The pain was most severe in the morning, when the patient first got out of bed, and in the evening, after he had been active all day. There was no history of trauma, and the patient denied other joint symptoms. He reported that acetaminophen 650 mg two or three times daily had been effective for managing his pain but no longer was. He tried ibuprofen 400 mg three times daily, and while it provided adequate pain relief, he was reluctant to begin nonsteroidal anti-inflammatory drug (NSAID) therapy for fear of gastrointestinal (GI) side effects. The patient had taken tramadol, hydrocodone, and oxycodone in the past but experienced troublesome lightheadedness.

The patient’s past medical history was significant for coronary artery disease and three prior myocardial infarctions (MIs), the most recent having been 2 years earlier. He was told to take aspirin 81 mg once daily to relieve his current pain. He denied any history of peptic ulcer disease, upper GI bleeding or other bleeding disorders, liver or kidney disease, chronic steroid use, and excessive alcohol intake.

On physical examination, the patient was able to walk without an assistive device but demonstrated a slightly antalgic gait. Hip internal rotation was painful and limited to 15° bilaterally. External rotation was 50° on the right side and 40° on the left side. Hip flexion and extension were within normal limits. Flexion-abduction-external rotation (FABER) provocative testing produced hip pain bilaterally. Results of a lower-extremity strength examination are indicated in Table 1. Sensation was intact to light touch. Examination of the patient’s knees demonstrated mild crepitus bilaterally, and results of a lumbar spinal examination were unremarkable.

**Table 1.** Lower-Extremity Strength*

	Hip Flexion	Hip Extension	Hip Abduction	Hip Adduction	Knee Extension	Knee Flexion	Ankle Dorsiflexion	Ankle Plantarflexion
R	5/5	5/5	4/5	4+/5	5/5	5/5	5/5	5/5
L	5/5	5/5	4+/5	4+/5	5/5	5/5	5/5	5/5

*Graded on a scale of 1 to 5, where 1 represents minimal strength and 5 represents maximal strength.

This patient met the American College of Rheumatology clinical criteria for OA of the hip [2], which was hip pain plus hip internal rotation of ≥15°, pain with hip internal rotation, morning stiffness of the hip for ≤60 minutes, and age >50 years *or* hip pain plus hip internal rotation of <15° and erythrocyte sedimentation rate (ESR) of ≤45 mm/hr or hip flexion of <115° if the ESR is not known.

### Treatment

The patient was concerned about long-term NSAID therapy because of potential GI side effects and was reluctant to retry opioid therapy since he had previously experienced lightheadedness. With the patient’s prior history of MIs, cyclooxygenase (COX)-2-specific inhibitors were ruled out as a potential therapy. After reviewing the benefits and relative risk reduction of GI adverse events provided when a proton pump inhibitor (PPI) is used in combination with a nonselective NSAID, his physician decided to start the patient on over-the-counter (OTC) naproxen 220 mg every 12 hours in conjunction with the PPI omeprazole 20 mg once daily. The patient was advised of the black box warnings regarding naproxen that are related to cardiovascular (CV) and GI adverse events [3], as well as other possible side effects. He also advised the patient to start a program of physical therapy and instructed him to continue taking acetaminophen 650 mg up to four times per day as needed.

When the patient returned to the office 4 weeks later, the patient had been in a physical therapy program for 3 weeks and was taking naproxen 440 mg in the morning and 220 mg at night, omeprazole 20 mg once daily, and acetaminophen 650 mg twice daily. He rated his pain on this regimen as a 5 on a ten-point scale in the left hip and a 2 in the right hip. He was given a straight cane by a physical therapist, which he found to be very helpful in alleviating some of his pain during walking. He reported no adverse GI effects and was pleased with his treatment and progress. He was able to tolerate physical therapy and was learning a home exercise program. A follow-up visit was scheduled for 2 months, during which time the patient will have completed his course of physical therapy; however, he will continue his home exercise program and adhere to his medication regimen.

## Discussion

While initial treatment for OA should include nonpharmacologic intervention, when such therapy provides insufficient pain relief, medications should be added while the non-pharmacologic therapy is continued. After careful assessment of risk factors and medical co-morbidities, initial pharmacologic management should begin with acetaminophen or a nonselective NSAID, such as naproxen or ibuprofen. While the nonselective NSAIDs should be used with caution because of their potential for GI side effects, results from a recent observational study showed that the addition of a PPI to NSAID therapy reduced the risk of peptic ulcer bleeding by 82% [4]. The study also showed that patients taking a nonselective NSAID and a PPI have the same incidence of NSAID-induced gastropathy as patients taking a COX-2-specific inhibitor. Currently, two fixed-dose combinations of an NSAID and a PPI are in development; naproxen/esomeprazole from AstraZeneca [5] and ibuprofen/famotidine from Horizon Therapeutics [6]. While both ibuprofen and naproxen are available in higher, prescription-strength doses as well as lower, OTC doses, the occurrence of adverse GI side effects in patients taking OTC doses of naproxen (up to 660 mg/day) has been shown to be comparable to, and in some cases lower than, the incidence of GI side effects in patients taking placebo [7]. Additionally, while COX-2-specific inhibitors have been linked to an increased risk of CV events [8], naproxen has been shown not to be associated with an increased risk of acute MI [9,10].

Another important issue that arises when initiating treatment for patients with OA who are already taking aspirin for cardioprotection is that certain NSAIDs may interfere with the antithrombotic benefits of aspirin through a competitive interaction with platelet COX-1. The co administration of ibuprofen and aspirin has been shown to antagonize aspirin’s irreversible platelet inhibition, indicating that the use of ibuprofen in patients at increased CV risk could limit the cardioprotective effects of aspirin [11,12]. *Post hoc* analysis of the TARGET (Therapeutic Arthritis Research and Gastrointestinal Event Trial) study showed that among patients at high CV risk already taking aspirin, the incidence of CV events was significantly higher in the subgroup taking ibuprofen but not in the subgroup taking naproxen when each was compared with lumiracoxib [13]. The concomitant administration of acetaminophen [11,14], or naproxen in prescription [15], or nonprescription doses [11], has not been shown to reduce the COX-1-mediated platelet inhibition induced by aspirin, suggesting that these two medications may be safer choices in patients at higher CV risk who are receiving aspirin therapy.

## Conclusions

The application of clinical data to decision making is critical in determining the optimal treatment regimen for patients with OA. The use of OTC naproxen in association with a PPI and low-dose aspirin was found to be effective in treating OA of the hip in this patient with CV and GI risk factors.

## Abbreviations

COX: cyclooxygenase
CV: cardiovascular
ESR: erythrocyte sedimentation rate
FABER: flexion-abduction-external rotation
GI: gastrointestinal
MI: myocardial infarction
NSAID: nonsteroidal anti-inflammatory drug
OA: osteoarthritis
OTC: over-the-counter
PPI: proton pump inhibitor
TARGET: Therapeutic Arthritis Research and Gastrointestinal Event Trial
